# EfficientIR-Det Towards Efficient and Accurate DETR for UAV Infrared Object Detection

**DOI:** 10.3390/s26103129

**Published:** 2026-05-15

**Authors:** Xiang Yang, Hanbin Li, Xiaolan Xie

**Affiliations:** 1 College of Computer Science and Engineering, Guilin University of Technology, Guilin 541006, China; 1994022@glut.edu.cn; 2 Guangxi Key Laboratory of Embedded Technology and Intelligent System, Guilin University of Technology, Guilin 541004, China

**Keywords:** infrared object detection, DETR, UAV, state space model, end-to-end detection

## Abstract

Infrared (IR) object detection on unmanned aerial vehicle (UAV) platforms is fundamentally challenged by low signal-to-noise ratios and extremely tight onboard computational budgets. Conventional CNNs lack sufficient global context, while Transformers suffer from quadratic complexity, hindering real-time deployment. To address these bottlenecks, we propose EfficientIR-Det, a lightweight end-to-end detector featuring a holistic optimization of the backbone, encoder, and sampling mechanisms. Specifically, we design a Partial Star Network (PSN) backbone that achieves implicit high-dimensional feature expansion via element-wise multiplication to amplify weak IR signals with minimal redundancy. Furthermore, a Hierarchical Mamba (HiMamba) encoder leverages selective state-space modeling to provide linear-complexity global enhancement with superior hardware efficiency. To refine cross-scale representations, we introduce an Adaptive Gated Sampling (AGS) module and a Hierarchical Sampling Strategy (HSS) to optimize feature fusion and sampling budget allocation toward dim-small targets. On HIT-UAV, EfficientIR-Det achieves 88.4% mAP@0.5, outperforming the RT-DETR-R18 baseline by 3.3 points while reducing FLOPs and parameters by 48.9% and 44.2%, respectively. On the larger-scale DroneVehicle dataset, it consistently leads with a 74.1% mAP@0.5 and a high inference speed of 140.8 FPS. Our results offer a promising research scheme for robust, real-time infrared perception on edge-constrained UAV platforms.

## 1. Introduction

Unmanned aerial vehicle (UAV) platforms play an increasingly vital role in modern target detection tasks due to their maneuverability, wide coverage, and low deployment costs [1,2]. Infrared (IR) imaging technology operates independently of ambient illumination and maintains stable performance in scenarios where visible light fails, making it an ideal choice for UAV-based reconnaissance and search and rescue [3,4]. However, UAV-based IR detection faces the following unique challenges: From an imaging perspective, targets often manifest as dim-small patches submerged in low signal-to-noise ratio backgrounds with weak contrast and minimal texture [5,6,7]. From a deployment perspective, the limited onboard computational resources of UAVs impose strict constraints on model parameters and FLOPs. Consequently, the central focus of current research has shifted towards achieving a robust accuracy–efficiency trade-off tailored for infrared thermal characteristics.

From a physical perspective, infrared small targets are characterized by extremely low signal-to-noise ratios, limited spatial extent, and weak intensity contrast against complex thermal backgrounds. These properties imply that effective detection requires both local enhancement of subtle intensity variations and the ability to aggregate weak responses over long-range contexts. However, conventional convolutions tend to smooth such weak signals, while heavy attention mechanisms are computationally prohibitive for UAV deployment. Therefore, a suitable solution should jointly enable lightweight local saliency amplification and efficient global dependency modeling.

Early IR target detection primarily relied on hand-crafted features such as Local Contrast Measure (LCM) [8] and background modeling [9,10]. While effective in controlled environments, these methods exhibit limited generalization under complex thermal clutter [11] due to their heavy dependence on manual prior tuning. The emergence of deep learning has revolutionized the field. Convolutional Neural Networks (CNNs), including Faster R-CNN [12] and the YOLO series [13], significantly improve robustness. However, the inherent local receptive field of convolutions often leads to the loss of subtle thermal signatures during downsampling.

In view of the above shortcomings, recent works have explored specialized feature enhancement. For instance, TAF-YOLO [14] integrates adaptive fusion at the pixel level to preserve small-object details, while CCSFuse [15] designs collaborative compensation to rectify typical IR “ghosting” artifacts and boundary diffusion caused by thermal radiation. Complementarity-aware fusion strategies like CFFDNet [16] have also been proposed to amplify differential features in weak IR signals.

Although Transformer-based paradigms like Swin-Transformer [17] and DINO [18] capture global dependencies, their quadratic computational complexity $O(N^{2})$ poses a severe bottleneck for real-time UAV processing. Even the state-of-the-art RT-DETR [19] and Deformable-DETR [20] face challenges in maintaining high-resolution feature integrity under stringent power budgets. Even advanced frameworks such as R2PLoc [21], which utilizes hierarchical semantic aggregation for geo-localization, or ship incremental recognition models [22] designed for open-world scenarios, often entail high parameter counts and computational overhead.

To address these limitations, we propose EfficientIR-Det, a lightweight framework optimized for the holistic IR detection pipeline. Our design is physically motivated by the need to enhance weak local responses and capture long-range dependencies under low-SNR infrared conditions. Specifically, we introduce the Partial Star Network (PSN) as a backbone. Inspired by the “star operation” [23], PSN applies element-wise multiplications to a subset of channels to enhance subtle local intensity variations, which is particularly suitable for weak and texture-less infrared targets. Furthermore, recognizing that the hardware-aware design of Mamba—incorporating parallel scans and kernel fusion [24]—overcomes the GPU utilization issues of traditional SSMs, we propose the Hierarchical Mamba (HiMamba) encoder. HiMamba achieves O(N) linear-complexity global modeling, effectively capturing long-range contextual relationships and facilitating weak signal accumulation under noisy infrared backgrounds. Additionally, an Adaptive Gated Sampling (AGS) module and a Hierarchical Sampling Strategy (HSS) are integrated to refine cross-scale feature propagation, ensuring that even the most inconspicuous thermal targets are accurately localized.

The main contributions of this paper are summarized as follows:

1. We design the Partial Star Network (PSN), which utilizes a channel-splitting strategy and star operations to maintain high-dimensional feature representation for texture-less IR targets with minimal computational cost.
2. We propose the Hierarchical Mamba (HiMamba) encoder, leveraging selective state space modeling [25] to achieve global feature enhancement at linear complexity, bypassing the quadratic bottleneck of standard attention [26] while ensuring hardware-efficient execution.
3. We introduce the Adaptive Gated Sampling (AGS) module, which employs a dual-branch architecture and channel-adaptive gating to enhance the propagation of weak IR features across the pyramid.
4. We develop a Hierarchical Sampling Strategy (HSS) that dynamically allocates sampling points for deformable attention based on scale-specific feature contributions, significantly improving localization accuracy.

## 2. Related Work

The study of UAV-based infrared (IR) target detection has gained significant traction as thermal sensors provide crucial signatures in low-visibility environments. In this section, we review the evolution of IR detection, the paradigm shift in end-to-end detectors, advances in lightweight architectures, and the emerging selective state-space models.

### 2.1. Infrared Target Detection in UAV Images

**Figure 1:**
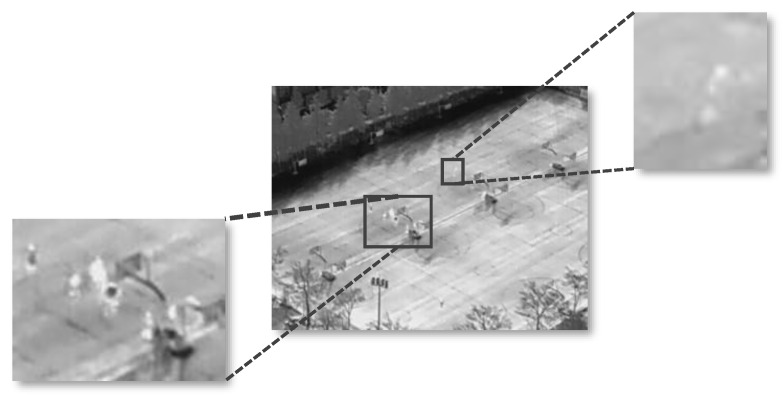
Infrared target from a drone’s view.

Infrared sensors on UAVs typically operate at high altitudes, resulting in targets that occupy extremely few pixels and lack distinctive color or texture.Infrared Target from a Drone’s View as Figure 1. Unlike generic object detection, UAV-IR detection must contend with severe thermal clutter and sensor noise [27].

Early research focused on background suppression and local contrast measures [28]. However, as noted by recent surveys, these hand-crafted priors fail in dynamic, complex scenes. Recent deep learning approaches have shifted towards specialized architectures. For instance, MDCE-Net [29] employs multi-dimensional cross-enhancement, while other works leverage super-resolution [30] or attention-based fusion [31,32] to recover weak signatures. Despite these advances, most current UAV-IR detectors rely on local convolutional operators, which struggle to capture the long-range thermal dependencies necessary for distinguishing dim targets from background noise. This motivates our design of a global–local balanced framework.

### 2.2. End-to-End Object Detectors

The DETR paradigm [33] revolutionized detection by eliminating the need for hand-crafted anchors and NMS via bipartite matching. To address the slow convergence of the original DETR, Deformable-DETR introduced multi-scale deformable attention, while DINO further improved performance through contrastive denoising and mixed query selection.

However, the computational cost of the Transformer encoder remains a major hurdle for UAV deployment. Swin-Transformer-based DETRs utilize window-based attention to reduce complexity, but they still exhibit higher overhead than their CNN-based counterparts like YOLOv11 [34] or YOLOv26 [35]. RT-DETR achieved real-time performance by employing a hybrid encoder, yet its attention layers still scale quadratically ($O(N^{2})$) with feature resolution. Our work aims to replace these heavy attention mechanisms with linear-complexity alternatives without sacrificing the end-to-end advantage.

### 2.3. Lightweight Network Design

Efficient architecture design is critical for resource-constrained UAV platforms. Beyond model compression [36,37], recent research focuses on efficient operators like Depthwise Convolutions [38], Channel Shuffle [39], and Partial Convolutions (PConv) [40]. A breakthrough in this direction is the Star Operation, which utilizes element-wise multiplications in a star-shaped topology to project features into a high-dimensional implicit space with negligible FLOPs. While effective in classification, the application of star operations to the specific task of IR small-target detection remains under-explored. Our proposed PSN backbone extends this concept to better capture subtle IR features.

### 2.4. State-Space Models

State-Space Models (SSMs), particularly Mamba, have emerged as a potent alternative to Transformers for long-sequence modeling. Unlike the $O(N^{2})$ complexity of self-attention, Mamba achieves O(N) linear complexity through input-dependent selective scans.

Crucially, Mamba’s efficiency stems from its hardware-aware design, which leverages parallel scan algorithms and kernel fusion to minimize memory I/O overhead on GPUs—a significant advantage over traditional RNN-based SSMs [41]. Following the success of Vision Mamba [42] and VMamba [43], several works have explored windowed or local scanning strategies [44] to enhance spatial modeling. In this paper, we build upon these hardware-efficient primitives to design the HiMamba encoder, specifically optimized for the high-resolution feature maps common in UAV-IR detection.

## 3. Methods and Methodology

**Figure 2:**
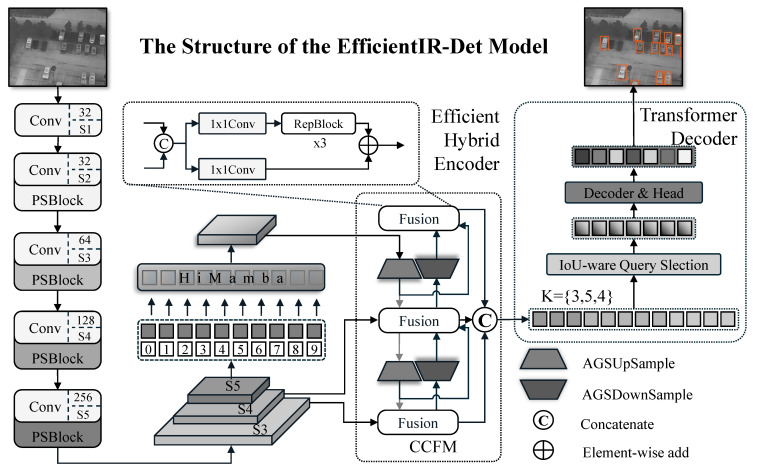
The structure of the EfficientIR-Det model. The PSN is composed of PSBlock and conventional layers, where the channel numbers for each stage are annotated. The decoder is featured by our HSS with an allocation strategy of {3, 5, 4}. Darker colors indicate deeper stages.

The overall architecture of EfficientIR-Det is illustrated in Figure 2. Given an input image, the Partial Star Network (PSN) first extracts multi-scale features $\left\{S_{2},S_{3},S_{4},S_{5}\right\}$ at strides {4,8,16,32}. The multi-scale features are then enhanced by an Efficient Hybrid Encoder. Following the design of RT-DETR, we apply global modeling only to the deepest level $S_{5}$ to strengthen semantic expressiveness. Unlike the original AIFI module in RT-DETR, we replace it with a Hierarchical Mamba (HiMamba) encoder. Built on state space models (SSM), HiMamba performs sequence modeling via a simplified selective scan mechanism—retaining only horizontal and vertical bidirectional scans—to capture long-range dependencies with linear complexity O(N).

The enhanced $S_{5}$ is fused with $S_{3}$ and $S_{4}$ by a Feature Pyramid Network (FPN) using an Adaptive Gated Sampling (AGS) module for cross-scale aggregation, yielding three output features $\left\{S_{3},S_{4},S_{5}\right\}$. Finally, the decoder aggregates information from the multi-scale features via deformable attention, where we introduce a Hierarchical Sampling Strategy (HSS): unlike standard RT-DETR that uses a uniform number of sampling points *K* = 4 for all scales, HSS assigns scale-aware sampling budgets [4,5,3] to $S_{3}$, $S_{4}$, and $S_{5}$, respectively, considering their resolutions and contributions to small-object detection. This reallocates a near-equal overall sampling budget while improving computational efficiency.

In summary, EfficientIR-Det attains an end-to-end light-weight design via the following: (i) a compact backbone that preserves compute, (ii) an efficient HiMamba encoder that preserves semantics, (iii) AGS for cross-scale information flow, and (iv) HSS that steers the decoder toward salient regions. The following sections provide details.

### 3.1. Partial Star Network (PSN)

The backbone is the primary engine for extracting hierarchical representations. For UAV-based infrared (IR) detection, the backbone must balance the amplification of faint thermal signatures with the strict computational limits of onboard hardware, as infrared targets are often weak, low-contrast, and easily submerged in noise. To achieve this, we propose the Partial Star Network (PSN), which leverages the “star operation” within a partial computation framework to achieve implicit feature expansion at a minimal cost.

#### 3.1.1. Motivation: Exploiting Feature Redundancy

**Figure 3:**
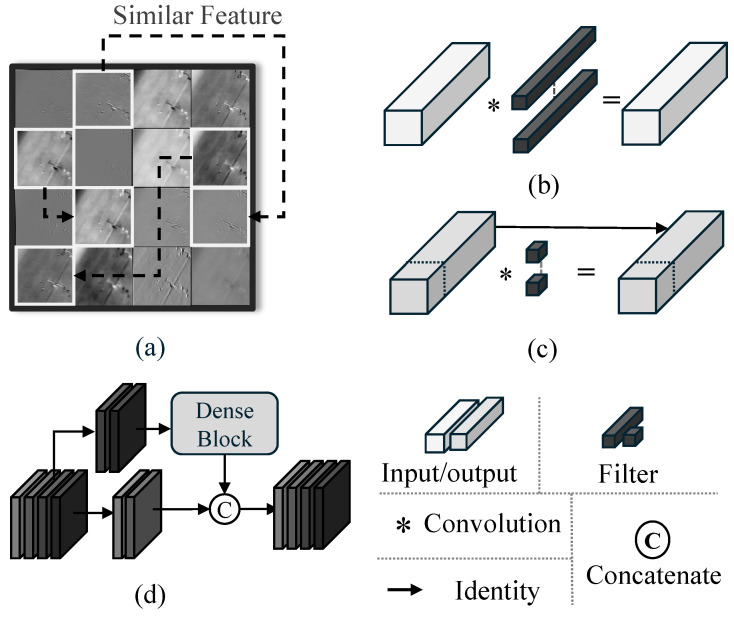
Illustration of feature redundancy and partial computation strategies in CNNs. (**a**) Redundant and highly similar feature maps in conventional CNNs, as motivated by GhostNet. (**b**) Standard convolution applied to all channels. (**c**) Partial convolution in FasterNet, where only a subset of channels is convolved while others follow identity mapping. (**d**) Cross Stage Partial connections in CSPNet that split and merge transformed and identity branches.

As illustrated in Figure 3, conventional CNNs suffer from significant feature redundancy, where many channels exhibit highly similar activation patterns [45]. (a) While standard convolutions (b) process all channels indiscriminately, recent efficient designs such as FasterNet [40] and CSPNet [46] utilize partial convolutions (c) or cross-stage branching (d) to reduce redundant FLOPs.

Inspired by these strategies, we argue that for IR images—which lack complex textures but which lack complex textures but rely on subtle intensity variations to distinguish targets from the background—it is sufficient to apply heavy nonlinear transformations to only a subset of channels.This allows the backbone to concentrate its “expressive budget” on the most informative features while maintaining a high-speed identity flow for stable background components.

#### 3.1.2. Star Operation for Infrared Feature Augmentation

To enhance the discriminability of dim targets without inflating the model, we utilize the Star Operation. By employing element-wise multiplication, this operation acts as an implicit polynomial kernel that lifts features into a high-dimensional nonlinear space.

Given an input *x*, we project it into two parallel branches $F_{1},F_{2}$ via 1×1 convolutions. The star operation is defined as follows:

(1) $$f_{\mathrm{star}}=\left(w_{1}^{⊤}x+b_{1}\right)\cdot \left(w_{2}^{⊤}x+b_{2}\right)=\sum_{i,j}w_{1}^{\left(i\right)}w_{2}^{\left(j\right)}x^{\left(i\right)}x^{\left(j\right)}+\ldots$$

By constructing second-order combinations of input channels, the star operation implicitly expands the feature dimension from *d* to $D_{\mathrm{implicit}}\approx d^{2}/2$. In IR scenarios, this provides the kernel-like expressivity needed to amplify subtle thermal variations against complex backgrounds without the parameter overhead of deeper stacks.

#### 3.1.3. Partial Star Block (PSB) Architecture

**Figure 4:**
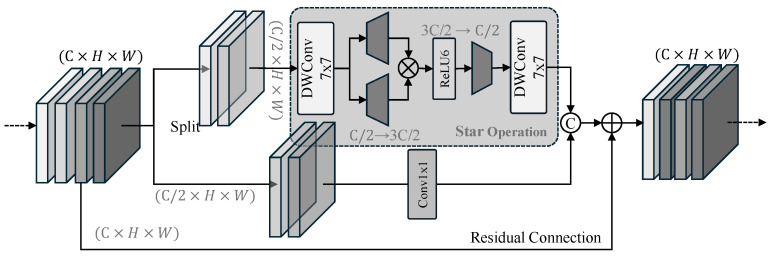
The structure of the Partial Star Block. The symbol “C” inside a circle denotes concatenation, the cross inside a circle denotes element-wise multiplication, and the plus sign inside a circle denotes element-wise addition.

The core unit of the proposed backbone is the Partial Star Block (PSB), as illustrated in Figure 4. Adhering to the partial computation principle, the input tensor $X\in R^{C\times H\times W}$ is partitioned into two functional paths to balance representation capacity with execution efficiency.

The upper branch, designated as the Star Path, is engineered to extract deep nonlinear patterns. It sequentially consists of a 7×7 depthwise convolution (DWConv) for local spatial modeling, followed by the Star Operation—which integrates two 1×1 projections, an element-wise multiplication, and a ReLU6 activation—to facilitate implicit dimensional expansion. A final 7×7 DWConv is then applied to refine these augmented features. Concurrently, the lower branch, or Direct Path, employs a lightweight 1×1 convolution to maintain a high-speed feature flow. This path is crucial for preserving the stable radiance intensity of the infrared scene, functioning as a residual-like identity mapping that effectively prevents the degradation of global contextual information.

Ultimately, the outputs from both paths are concatenated and subsequently integrated with the original input via a residual connection. This dual-path architecture ensures that the Partial Star Network (PSN) remains highly sensitive to weak targets while maintaining competitive inference latency for real-time applications.

### 3.2. Hierarchical Mamba Encoder (HiMamba)

We replace the AIFI module with a Hierarchical Mamba (HiMamba) encoder. HiMamba is built on state space model (SSM) for sequence modeling, whose key advantage lies in its linear memory complexity: it does not need to explicitly construct and store attention matrices. Instead, long-range dependencies are captured implicitly through recursive state transitions.

The selective state space model Mamba employs input-dependent parameterization and hardware-aware algorithm design. This enables it to achieve Transformer-level modeling capability while maintaining linear complexity. Gu and Dao [25] show that, although Mamba may have slightly higher theoretical FLOPs than Transformers, its sequential memory access pattern and avoidance of large explicit matrices yield faster inference on real hardware.

#### 3.2.1. Performance Bottlenecks of AIFI

RT-DETR introduces the Attention-based Intrascale Feature Interaction (AIFI) module on the $S_{5}$ feature level to enhance deep semantic representations. AIFI adopts a standard multi-head self-attention (MHSA) mechanism and performs global modeling over all spatial locations of the feature map. For an input image of size 640 × 640, the $S_{5}$ feature map has size 20 × 20 (sequence length *N* = 400), and AIFI has to construct and process a 400 × 400 attention matrix.

FlashAttention [24] reveals a crucial fact: in practice, the bottleneck of attention mechanisms lies in *memory bandwidth* rather than *arithmetic intensity*. Through an I/O complexity analysis, Dao et al. demonstrate that standard MHSA requires $O(N^{2})$ accesses to high-bandwidth memory (HBM), which leads to significant latency on GPUs. The total number of HBM accesses can be written as $O(NC+N^{2})$. Under the configuration N=400, C=256, the quadratic term $N^{2}=$ 160,000 far exceeds the linear term NC= 102,400, making the reads and writes of the attention matrix the dominant cost.

**Figure 5:**
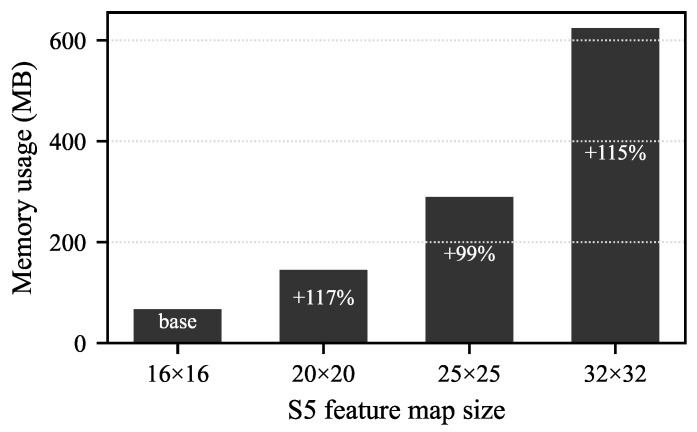
Memory usage of AIFI at different input resolutions; the annotated growth rates are measured relative to the previous resolution. Even at the low-resolution S5 scale, the memory consumption still grows quadratically.

The quadratic memory footprint of the attention matrix grows rapidly with the sequence length *N*. As shown in Figure 5, when the $S_{5}$ feature map is upsampled from 16×16 to 32×32, the GPU memory consumption of AIFI increases sharply from 67.1 MB to 623.8 MB, an overall increase of about 9.3×. The increases between adjacent scales are all close to or above 1× (+117%, +99%, +115%), which is far larger than the linear growth in the feature map side length. This observation indicates that the memory cost of AIFI does not grow linearly with the feature size, but it is heavily affected by the $O(N^{2})$ accesses of the attention matrix. As the $S_{5}$ scale increases, the $N^{2}$ term quickly dominates the total memory traffic, making attention-matrix I/O the main performance bottleneck at this level.

#### 3.2.2. The Architectural Design of HiMamba

To overcome the quadratic complexity of the self-attention mechanism in the standard Transformer encoder, while addressing the need to aggregate weak infrared signals under low-SNR conditions, we propose the Hierarchical Mamba (HiMamba) encoder as a linear-complexity alternative to the AIFI module in RT-DETR. HiMamba leverages the Selective State Space Model (SSM) to capture global dependencies while maintaining hardware efficiency.

##### Mathematical Foundation

The core of HiMamba is the discretized selective SSM. Given an input sequence $x_{k}\in R^{D}$, the system evolves through a hidden state $h_{k}\in R^{N}$ as follows:

(2) $$\begin{array}{cc} h_{k} & =\bar{A}\;h_{k-1}+\bar{B}\;x_{k},\\ y_{k} & =Ch_{k}+\bar{D}\;x_{k}, \end{array}$$

where $\bar{A}=e^{\Delta A}$ and $\bar{B}={\left(\Delta A\right)}^{-1}\left(e^{\Delta A}-I\right)\Delta B$ represent the discretized parameters using the Zero-Order Hold (ZOH) method. Unlike traditional SSMs, Mamba makes Δ,B,C functions of the input *x*, allowing the model to adaptively “select” relevant information. This selective mechanism allows the model to progressively integrate consistent target responses while suppressing stochastic variations, which is particularly beneficial for infrared scenes where targets are weak and easily obscured by noise. Furthermore, its hardware-aware implementation—utilizing parallel scan algorithms and kernel fusion—ensures that the recursive update avoids memory I/O bottlenecks, achieving significantly higher GPU utilization than RNNs.

##### Structural Decomposition of HiMamba

**Figure 6:**
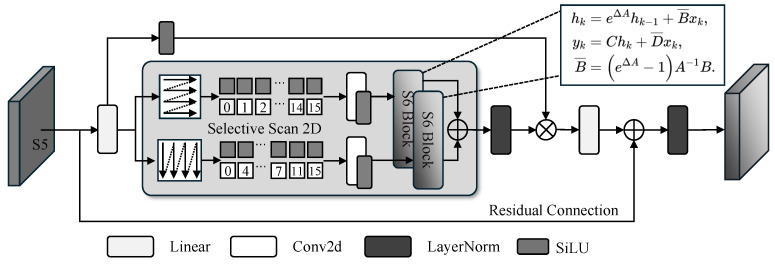
Overall architecture of the Hierarchical Mamba Encoder. The cross inside a circle denotes element-wise multiplication, and the plus sign inside a circle denotes element-wise addition.

As illustrated in Figure 6, the HiMamba block adopts a dual-branch gated architecture to enhance feature modulation through state-space modeling. Specifically, given an input feature map derived from the $S_{5}$ stage, a LayerNorm layer is first employed to stabilize the distribution. The normalized features are then bifurcated into two parallel computational streams via linear projections.

In the primary stream, denoted as the Selective Branch, a 3×3 depthwise convolution (DWConv) is initially applied to capture local spatial priors. This is followed by the Selective Scan 2D (SS2D) module, where we simplify the conventional cross-scan mechanism [42] by retaining only bidirectional horizontal and vertical scanning trajectories [47]. This strategic simplification aims to achieve an optimal balance between representation capacity and computational efficiency; a comprehensive empirical justification for this design choice is further elaborated in the experimental section. This SS2D core performs selective integration along the spatial axes, thereby enabling long-range dependency modeling that helps accumulate weak target responses across spatial contexts.Concurrently, the auxiliary Gating Branch processes the features through a SiLU activation to generate a modulation signal.

The outputs from both branches are subsequently fused via element-wise multiplication (⊗), forming a structure analogous to a Gated Linear Unit (GLU). This configuration enables the network to adaptively suppress background clutter while selectively amplifying the responses of dim-small infrared targets under low signal-to-noise conditions. Finally, a linear projection is utilized to restore the channel dimensionality, followed by a residual connection to ensure a stable gradient flow across the architecture.

This design enables HiMamba to model long-range dependencies efficiently while remaining well-suited for weak signal detection in infrared imagery.

##### Efficiency Benchmarking and Analysis

**Figure 7:**
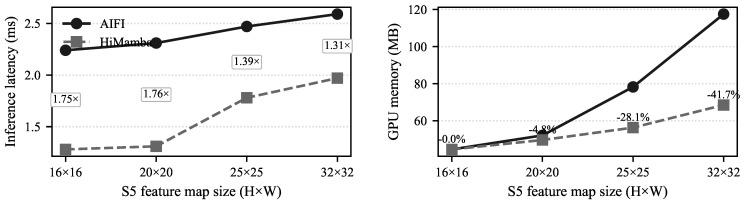
Impact of HiMamba and AIFI on inference speed and GPU memory usage at different resolutions.

We further compare HiMamba with the original AIFI module under different feature map resolutions, as shown in Figure 7. As the spatial resolution increases from 16×16 to 32×32, the latency of AIFI grows noticeably, which is consistent with the quadratic cost of self-attention. In contrast, HiMamba increases more gradually. For instance, at 20×20, it runs about 1.76× faster. This behavior is mainly due to the linear scan mechanism in Mamba, which avoids constructing large attention matrices and keeps computation more stable as the sequence length grows.

A similar pattern can be observed in memory usage. The memory consumption of AIFI rises quickly with resolution, while HiMamba remains more controlled. At 32×32, the memory usage is reduced by about 41.7%, making it easier to process higher-resolution feature maps under limited GPU resources. This difference comes from the fact that HiMamba does not rely on pairwise attention maps and therefore avoids the main source of quadratic overhead.

In the context of infrared detection, such behavior is useful because global context can be retained without introducing excessive computational cost, which helps maintain stable responses for weak targets in noisy backgrounds.

### 3.3. Adaptive Gated Sampling (AGS) Module

**Figure 8:**
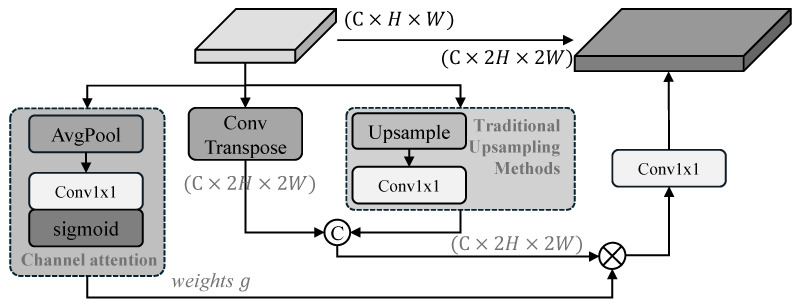
Diagram of the Adaptive Gated Sampling (AGS) module. The symbol “C” inside a circle denotes channel-wise concatenation, and the cross inside a circle denotes element-wise multiplication.

In UAV-based infrared scenarios, the inherent low resolution and fuzzy boundaries of targets make them highly sensitive to information loss during cross-scale propagation. Conventional sampling methods in the feature pyramid—such as nearest-neighbor interpolation or strided convolutions—often introduce blurring or aliasing, further degrading localization accuracy for dim-small targets [48,49]. To mitigate this, we propose the Adaptive Gated Sampling (AGS) module (Figure 8) to replace the vanilla sampling layers in the Neck of the baseline RT-DETR. AGS integrates complementary sampling branches with a lightweight channel-wise gating mechanism to preserve crucial thermal signatures.

#### 3.3.1. Complementary Dual-Branch Architecture

As illustrated in Figure 8, the AGS module adopts a parallel two-branch design to balance learnable feature reconstruction with deterministic spatial priors.

For upsampling, AGS adopts a dual-branch design. One branch uses a 2×2 transposed convolution with stride 2 to learn task-specific kernels, which helps recover sharper target boundaries. The other branch relies on bilinear interpolation followed by a 1×1 convolution, providing a smoother structural reference and mitigating checkerboard artifacts. The two branches complement each other by balancing detail restoration and structural stability.

For downsampling, a similar dual-path strategy is employed. One branch applies a 3×3 strided convolution to aggregate higher-level semantic information, while the other branch consists of a 2×2 max pooling layer followed by a 1×1 convolution. In infrared imagery, max pooling tends to preserve local peak responses, which is beneficial for retaining the intensity of small targets against cluttered backgrounds.

The outputs of both branches are concatenated along the channel dimension to fuse the diverse sampling responses.

#### 3.3.2. Adaptive Channel-Wise Gating

To dynamically refine the fused features, AGS applies a global gating mechanism. First, a channel descriptor is extracted via Global Average Pooling (AvgPool). This descriptor is then processed by a 1×1 Conv and a Sigmoid activation to generate gating weights g∈[0,1], which represent the importance of each sampling channel.

The final refined feature $F_{out}$ is formulated as follows:

(3) $$F_{out}={\mathrm{Conv}}_{1\times 1}\left(\left[{\mathrm{Branch}}_{1}\;∥\;{\mathrm{Branch}}_{2}\right]⊗g\right)$$

where ∥ denotes concatenation and ⊗ denotes element-wise multiplication. This content-aware gating allows the module to emphasize the sharpened features from the learnable branch for target regions, while favoring the smooth baseline for background areas, thereby significantly improving the signal-to-clutter ratio (SCR).

### 3.4. Hierarchical Sampling Strategy (HSS)

**Figure 9:**
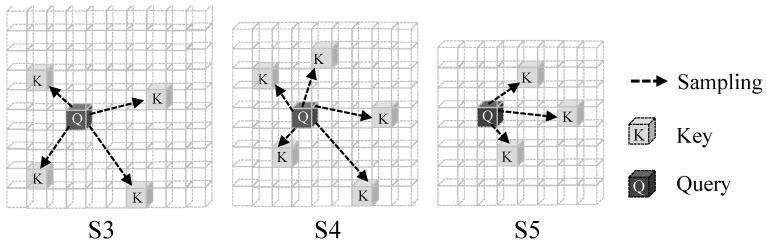
Illustration of the proposed Hierarchical Sampling Strategy (HSS). For a given query (Q), deformable attention samples different numbers of key locations (K) on each feature level: dense sampling on the high-resolution S3 map, moderate sampling on S4, and sparse sampling on S5. The total number of samples per query is kept fixed, while the sampling budget is redistributed according to the information density of each scale.

In the DETR decoder, deformable attention aggregates multi-scale features by sampling *K* locations around a reference point *p*. The standard configuration employs a uniform sampling budget (K=4) for all feature levels. However, this ignores the inherent information density disparity across the feature pyramid, especially in UAV-based infrared (IR) scenarios where small targets primarily rely on high-resolution spatial details. To optimize the allocation of computational resources, we propose the Hierarchical Sampling Strategy (HSS) (Figure 9).

#### 3.4.1. Information-Driven Resource Allocation

From an information-theoretic perspective, the contribution of each feature level to small-target localization is non-uniform. The $S_{3}$ level (stride = 8) contains fine-grained spatial cues, while $S_{5}$ (stride = 32) provides coarse semantic context.

We formulate the multi-scale feature aggregation as follows:

(4) $$y=\sum_{l=1}^{3}\sum_{k=1}^{K_{l}}w_{lk}\cdot \phi \left(x^{l},p_{l}+\Delta p_{lk}\right),$$

where $K_{l}$ is the number of sampling points for level *l*. Instead of the vanilla uniform setting ($K_{1}=K_{2}=K_{3}=4$), HSS redistributes the fixed total budget ($\sum K_{l}=12$) based on the scale-specific information density. In our implementation, we adopt a [4, 5, 3] configuration for ($S_{3},S_{4},S_{5}$). By reducing the redundant sampling in the low-resolution $S_{5}$ and augmenting the $S_{4}$ budget, the model enhances its ability to capture targets that span across medium-to-high resolutions, which is critical for the “dim-small” characteristics of infrared signatures.

#### 3.4.2. Zero-Cost Optimization

As illustrated in Figure 9, HSS reallocates the sampling points without introducing additional learnable parameters or increasing the total FLOPs. The total number of sampling operations $N_{\mathrm{sample}}$ remains constant:

(5) $$N_{\mathrm{sample}}=L_{\mathrm{dec}}\times N_{\mathrm{query}}\times \sum_{l=1}^{3}K_{l}=21,600,$$

where $L_{\mathrm{dec}}=6$ and $N_{\mathrm{query}}=300$.

This strategy serves as a task-specific prior. By shifting the sampling density toward higher-resolution levels, HSS effectively improves localization precision for weak targets. Unlike dynamic sampling mechanisms that require extra sub-networks, HSS achieves a competitive performance gain with zero deployment overhead, making it an ideal design choice for resource-constrained UAV edge devices.

## 4. Experiments

### 4.1. Experimental Settings

#### 4.1.1.  Datasets

**Figure 10:**
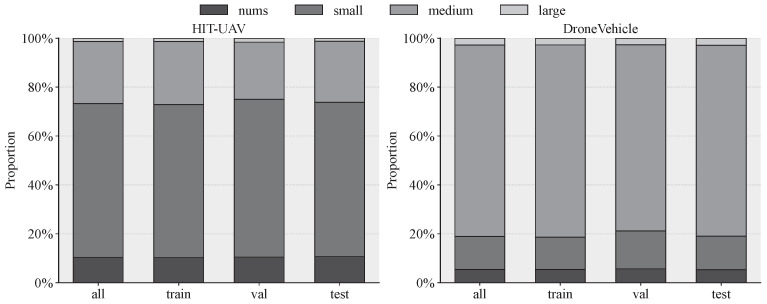
Comparison of target size distributions between the HIT-UAV and DroneVehicle datasets.

We evaluate the proposed method on two specialized drone-based benchmarks: HIT-UAV [50] and DroneVehicle [51]. The distribution of target scales for both datasets is illustrated in Figure 10.

The HIT-UAV dataset is a high-altitude infrared object detection benchmark containing 2898 images captured at altitudes from 60 m to 130 m. It features four categories: *Person*, *Car*, *Bicycle*, and *OtherVehicle*. As shown in Figure 10, small objects (area <32^2^ pixels) account for approximately 70% of the 24,751 annotations, making it a small object-dominated benchmark for infrared UAV detection. In our implementation, images are resized to 640×640 and pre-processed by mixing IDTransformer [52] denoised images with edge-contrast enhanced images.

The DroneVehicle dataset is a large-scale RGB-Infrared benchmark comprising 28,439 image pairs (56,878 images total) captured across diverse urban scenarios. In this study, we focus exclusively on the infrared (IR) modality to evaluate the model’s robustness in pure thermal imaging scenarios. As quantified in Figure 10, the dataset exhibits a balanced distribution of scales compared to HIT-UAV. Our experiments utilize the infrared subset, which contains 500,515 annotated instances: *Car* (428,083), *Truck* (25,960), *Freight car* (17,172), *Bus* (16,592), and *Van* (12,708).

#### 4.1.2. Evaluation Metrics

We evaluate the models in terms of detection accuracy, model complexity, and inference speed.

##### Detection Accuracy

For classification performance, we adopt precision and recall:

(6) $$\mathrm{Precision}=\frac{\mathrm{TP}}{\mathrm{TP}+\mathrm{FP}},\;\mathrm{Recall}=\frac{\mathrm{TP}}{\mathrm{TP}+\mathrm{FN}},$$

where TP, FP, and FN denote true positives, false positives, and false negatives, respectively.

For object detection, we use Average Precision (AP), computed as the area under the precision–recall curve. We report mAP@0.5 and mAP@0.5:0.95, corresponding to AP at a single IoU threshold of 0.5 and the average AP over IoU thresholds from 0.5 to 0.95 with a step of 0.05. Following the COCO evaluation protocol, ${\mathrm{AP}}_{s}$ denotes the average precision for objects with a pixel area smaller than $32^{2}$. Unless otherwise specified, mAP denotes the mean AP over all *k* classes:

(7) $$\mathrm{mAP}=\frac{1}{k}\sum_{i=1}^{k}{\mathrm{AP}}_{i}.$$

##### Complexity Metrics

Model complexity is measured by FLOPs and the number of parameters (Params). FLOPs denote the total number of floating-point operations in a forward pass, and Params denote the total number of trainable weights. Both are reported in GFLOPs and millions, respectively.

##### Speed Metric

Inference speed is measured by latency, defined as the average time to process a single image:

(8) $$\mathrm{Latency}=\frac{1}{M}\sum_{i=1}^{M}t_{i},$$

where $t_{i}$ is the inference time for the *i*-th image and *M* is the number of test images. Latency is reported in milliseconds (ms) on the target hardware after a short GPU warm-up.

#### 4.1.3. Implementation Details

All experiments are conducted on an Ubuntu 22.04.1 system equipped with an NVIDIA GeForce RTX 3090 GPU (24 GB VRAM) and an Intel 14-core CPU (3.7 GHz). The models are implemented using PyTorch 2.2.2 with CUDA 11.2 support, as detailed in Table 1.

##### Training Setup

All images are resized to 640×640 resolution as input. We use the AdamW optimizer with an initial learning rate of $1\times 10^{-4}$, momentum of 0.9, and weight decay of $1\times 10^{-4}$. The models are trained with a batch size of 16 and 8 data loading workers. A cosine annealing schedule is adopted for learning rate decay, with a warm-up period during the first 10 epochs. Regarding the training duration, we train the models for 300 epochs on the HIT-UAV dataset, while 100 epochs are utilized for the DroneVehicle dataset to ensure efficient convergence across different data scales. Data augmentation consists of random horizontal flipping with a probability of 0.5. Following RT-DETR, Varifocal Loss is used for classification, while L1 loss and GIoU loss are employed for bounding box regression to ensure a fair comparison.

##### Inference Setup

Inference performance is evaluated in the TensorRT 10.13.3 environment. All models are converted to FP16 precision to simulate real deployment. Latency is measured with a batch size of 1; after 100 warm-up iterations, the final latency is averaged over 1000 inference runs on the test set. The detailed definitions of the evaluation metrics can be found in Section 4.1.2.

### 4.2. Comparative Experiments

We evaluate EfficientIR-Det against mainstream detectors, including CNN-based models and end-to-end DETR-based detectors, across two diverse datasets: HIT-UAV (Table 2) and DroneVehicle (infrared) (Table 3).

Comparison with CNN-based models. Classical detectors like RetinaNet and Faster R-CNN exhibit suboptimal performance in UAV-IR scenarios due to high computational overhead and low precision. In contrast, our model demonstrates a superior accuracy–efficiency profile compared to the latest YOLO series. On HIT-UAV, EfficientIR-Det achieves 88.4% $mAP_{50}$, surpassing YOLOv11m by 0.5% while utilizing only 43.0% of its FLOPs (29.1 G vs. 67.7 G) and 55.5% of its parameters. While YOLOv26m maintains a higher peak throughput (up to 161.0 FPS), our model provides a more balanced trade-off, particularly on DroneVehicle where it delivers competitive precision (74.1% $mAP_{50}$) at a significantly lower parameter count (11.1 M vs. 20.3 M).

Comparison with DETR-based models. EfficientIR-Det significantly outperforms the RT-DETR family and other modern end-to-end models. Compared to the baseline RT-DETR-R18, our architecture achieves a substantial precision leap of 3.3% and 1.7% $mAP_{50}$ on HIT-UAV and DroneVehicle, respectively. Notably, on HIT-UAV, it even surpasses the much larger RT-DETR-L in both accuracy (88.4% vs. 85.5%) and inference speed (144.7 vs. 123.0 FPS). This consistent superiority across both datasets validates that our coordinated design of PSN and HiMamba effectively addresses the heavy computational burden typical of standard DETR-based models.

**Figure 11:**
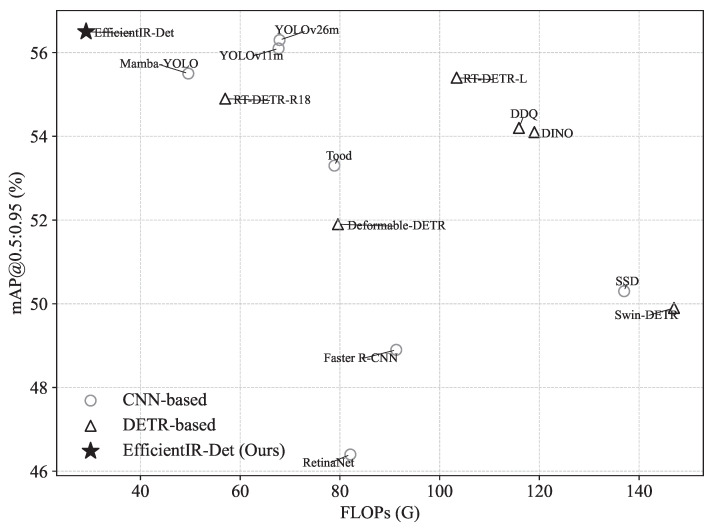
Scatter plot of computation cost versus accuracy for all compared models.

Operational reliability. In UAV surveillance, high precision and recall are critical to minimizing human verification. EfficientIR-Det achieves the highest precision (91.4% on HIT-UAV and 77.8% on DroneVehicle) and exceptional recall compared to other DETR-based models. As visualized in Figure 11, our model consistently occupies the upper-left region of the accuracy–efficiency map, confirming its robustness and practical utility for real-time edge deployment.

### 4.3. Inference Latency Analysis

**Figure 12:**
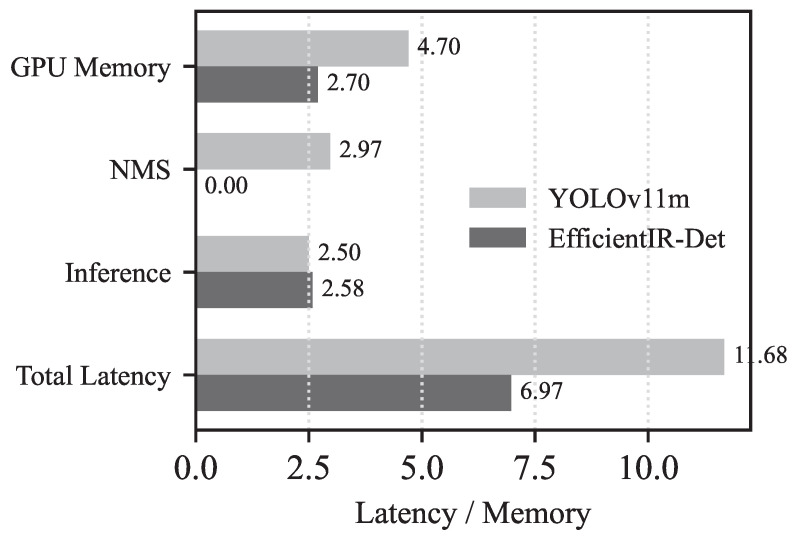
Detailed comparison of inference speed between our method and YOLOv11m.

To evaluate its efficiency, we break down the inference pipeline of EfficientIR-Det and compare it with YOLOv11m. Figure 12 illustrates the time consumption of each stage. In terms of pure model inference, the two detectors exhibit comparable latency: EfficientIR-Det achieves 2.58 ms, while YOLOv11m records 2.50 ms, with a marginal difference of only 0.08 ms. This indicates that, with the lightweight designs of PSN and HiMamba, EfficientIR-Det attains a YOLO-level execution speed while operating at merely 43.0% of its FLOPs (29.1 G vs. 67.7 G).

A substantial difference arises in the post-processing stage. YOLOv11m requires 2.97 ms for non-maximum suppression (NMS) to suppress redundant bounding boxes, resulting in a total post-processing overhead of 6.14 ms, which accounts for 52.6% of its overall latency. In contrast, EfficientIR-Det—as an end-to-end detector—leverages bipartite matching during training to enforce one-to-one predictions and therefore eliminates NMS during inference. Its post-processing consists only of a confidence-based Top-100 selection, costing merely 0.41 ms. This leads to a reduction of 5.73 ms in post-processing time, which nearly accounts for the entire latency advantage (4.71 ms) over YOLOv11m, highlighting the inherent benefits of end-to-end detection paradigms in simplifying the inference pipeline.

Regarding GPU memory usage, EfficientIR-Det occupies only 2.70% of GPU memory, compared with 4.70% for YOLOv11m—a 42.6% reduction. This memory advantage stems from the lightweight PSN backbone, which reduces intermediate feature map widths, and the linear-complexity HiMamba encoder, which avoids explicit storage of attention matrices. Lower memory usage enables larger batch sizes or multi-model concurrent inference on the same GPU, which is particularly valuable in multi-stream UAV surveillance applications.

Overall, EfficientIR-Det achieves a 40.3% improvement in total latency and a 42.6% reduction in memory usage compared with YOLOv11m, while exhibiting only a minor performance drop of 0.6 points. These results demonstrate that the proposed method achieves an excellent accuracy–efficiency trade-off, making it highly suitable for resource-constrained infrared object detection scenarios.

### 4.4. Ablation Studies

We conduct comprehensive ablation studies on the HIT-UAV and DroneVehicle datasets to verify the effectiveness of each proposed module and the superiority of our backbone design.

#### 4.4.1. Incremental Component Analysis

To systematically evaluate the performance–efficiency trade-offs of our proposed modules, we conduct incremental ablation studies on the HIT-UAV and DroneVehicle datasets, as detailed in Table 4 and Table 5.

The baseline performance 85.1% $mAP_{50}$ on HIT-UAV and 72.4% on DroneVehicle reveals a fundamental limitation of standard convolutional backbones in infrared (IR) scenarios: the inherent inductive bias of deep-stacked convolutions tends to “smooth out” weak intensity variations, causing small targets to vanish into complex thermal clutter. Substituting the ResNet-18 backbone with our PSN precipitates the most significant precision leap, boosting mAP@0.5 by 1.2% to 1.3% across both datasets while simultaneously slashing FLOPs by 55.4%. Physically, this underscores the advantage of the Star Operation; its non-linear multiplicative interaction acts as a latent feature amplifier, compensating for the characteristic texture-sparsity of IR signatures by expanding the representational dimension without the computational cost of additional layers.

The individual integration of the HiMamba(HiM) encoder highlights a distinct hardware-aware advantage. While maintaining competitive accuracy, it significantly pushes throughput—reaching 141.6 FPS on HIT-UAV and 139.3 FPS on DroneVehicle—validating its efficiency in modeling the long-range thermal dependencies typical of diffused heat signatures. When coupled with PSN, the PSN+HiM configuration achieves a Pareto-optimal result, yielding peak throughputs of 148.8 FPS and 147.4 FPS, respectively. This synergy suggests a functional division: PSN recalibrates local saliency, while HiMamba’s linear-complexity recursion aggregates these weak responses across the global spatial domain, effectively suppressing stochastic background noise that often misleads local-only detectors.

The subsequent introduction of the AGS neck and HSS head addresses the signal preservation challenge throughout the detection pipeline. AGS operates as a learnable signal-to-noise ratio (SNR) filter, raising mAP and notably improving Recall by ensuring that inconspicuous thermal targets are not suppressed during cross-scale fusion. Finally, the full ensemble (EfficientIR-Det) achieves peak performance, with a 3.3% and 1.7% overall $mAP_{50}$ gain over the respective baselines. These improvements, achieved alongside a substantial throughput increase, confirm that our architecture is a physically-grounded response to the unique constraints of UAV-based infrared imaging.

#### 4.4.2. Backbone Network Comparison

To evaluate the specific effectiveness of PSN, we compare it with the standard RT-DETR backbones (ResNet-18 and ResNet-L) as well as several recent lightweight architectures. The results on the HIT-UAV dataset are summarized in Table 6.

The baseline RT-DETR-R18 achieves 85.1% mAP@0.5, but its 57.0 G FLOPs and 19.9 M parameters pose significant challenges for real-time edge deployment. While the larger RT-DETR-L improves mAP@0.5:0.95 to 55.4%, its computational cost nearly doubles (103.4 G FLOPs), making it impractical for resource-constrained UAV platforms.

The comparison with specialized lightweight backbones reveals a prevalent trade-off between accuracy and efficiency in the infrared domain. For instance, FasterNet reduces FLOPs to 28.5 G via partial convolution, but its mAP@0.5 drops to 83.4% (−1.7 pp) due to the loss of weak IR features during aggressive downsampling. Similarly, StarNet, despite its efficient star operation, only attains 82.3% mAP, suggesting that its generic architecture is not fully optimized for low-SNR infrared imagery. Other models like EfficientViT and MobileNetV4 also show noticeable performance degradation, particularly in terms of Recall, indicating an insufficient capability for small-object capture.

In contrast, our PSN achieves a superior balance, reaching 86.4% mAP@0.5 (+1.3 pp over baseline) while reducing parameters and FLOPs by more than 50% (9.6 M and 25.4 G, respectively). Notably, PSN maintains the highest Precision (90.4%) and the lowest inference latency (7.53% ms, or 132.8 FPS). These results demonstrate that the integration of the partial computation paradigm with the polynomial kernel effect of the star operation successfully retains high-dimensional nonlinear expressiveness with minimal overhead. Consequently, PSN serves as an optimized backbone specifically suited for the texture-less and dim-small characteristics of infrared UAV imagery.

#### 4.4.3. Ablation of the HiMamba Encoder

To examine the effect of high-level feature interaction and the scanning design in HiMamba, we compare four encoder settings, including the interaction-free variant (w/o AIFI), the standard Transformer-based AIFI, the four-way scanning variant of HiMamba, and the proposed HiMamba encoder. The results are shown in Table 7.

Removing AIFI leads to a noticeable accuracy drop. The w/o AIFI variant obtains the highest inference speed of 151.7 FPS, but its mAP_50_ and mAP_50:95_ decrease to 83.9% and 54.2%, respectively. This result suggests that feature interaction at the high-level stage is still useful for infrared UAV target detection, where targets are often small and lack rich texture cues.

Compared with the Transformer-based AIFI, the proposed HiMamba achieves the same mAP_50_ of 85.1% and improves the mAP_50:95_ from 54.9% to 55.1%. Meanwhile, its inference speed increases from 127.1 FPS to 147.7 FPS. Although the recall of HiMamba is slightly lower than that of AIFI, it obtains the highest precision among all variants. These results indicate that HiMamba can maintain competitive detection accuracy while offering better inference efficiency.

We further introduce a four-way scanning variant, namely HiMamba-4way, to evaluate whether additional scanning directions can improve the modeling of spatial relationships. HiMamba-4way achieves 84.9% mAP_50_ and 55.1% mAP_50:95_, which are close to the results of the proposed HiMamba. However, its recall decreases from 82.5% to 82.2%, and its inference speed drops from 147.7 FPS to 131.6 FPS. This comparison shows that the additional scanning directions do not lead to a clear accuracy gain in our setting, but introduce extra computational cost. Therefore, we adopt the bidirectional scanning design in HiMamba, which provides a more favorable balance between accuracy and speed.

#### 4.4.4. Comparison of Feature Fusion Modules

To evaluate the specific superiority of our Adaptive Gated Sampling (AGS) module, we compare it with several representative feature fusion and sampling strategies, including SBA, WaveletPool, wConv, and GCCConv. The standard concatenation-based fusion is used as the baseline. The results are summarized in Table 8.

The baseline method achieves 86.7% mAP@0.5 and 55.1% mAP@0.5:0.95. Among the alternative approaches, WaveletPool and wConv exhibit performance degradation, yielding only 82.7% and 85.1% mAP@0.5, respectively. Their mAP@0.5:0.95 scores further confirm this downward trend, dropping to 52.1% and 53.5%. While GCCConv shows competitive results on the mAP@0.5:0.95 metric (54.7%), it falls short on mAP@0.5 (84.8%) compared to the baseline. SBA maintains relatively balanced performance but still underperforms the baseline across all primary metrics.

In contrast, our proposed AGS achieves the highest overall performance, reaching 88.0% mAP@0.5 (+1.3 pp over baseline) and 56.0% mAP@0.5:0.95 (+0.9 pp). More importantly, AGS demonstrates a substantial lead in Precision (91.0%) and Recall (85.9%), outperforming all competing methods. This confirms that the gated dual-branch architecture effectively mitigates the information loss common in traditional sampling, significantly enhancing the signal-to-clutter ratio for infrared small targets. These results empirically validate that AGS provides the most effective cross-scale feature communication for the UAV-based infrared detection task.

#### 4.4.5. Ablation Analysis of HSS

**Figure 13:**
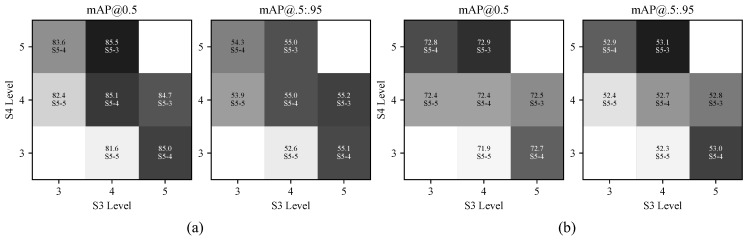
Heatmap analysis of the HSS module on the (**a**) HIT-UAV and (**b**) DroneVehicle datasets. The horizontal and vertical axes correspond to the S3 and S4 layers, respectively, while the value of S5 is annotated within each box. Darker colors indicate higher values in the heatmap.

To evaluate the effect of the proposed Hierarchical Sampling Strategy (HSS), we compare seven budget allocation strategies under the same constraint of $K_{\mathrm{total}}=12$. The uniform allocation [4,4,4] is used as the baseline. The quantitative results and the corresponding heatmaps on the two benchmarks are shown in Table 9 and Figure 13.

The results indicate that the allocation of the sampling budget has a clear influence on detection performance. When more samples are assigned to the low-resolution level $S_{5}$, such as the [3,4,5] setting, the performance decreases on both datasets, with ${\mathrm{mAP}}_{50}$ dropping to 82.4% on HIT-UAV and 72.4% on DroneVehicle. This suggests that excessive sampling at high-stride feature levels brings limited additional information and may reduce the model’s ability to preserve fine localization cues for small infrared targets.

A consistent observation across the two datasets is that the intermediate level $S_{4}$ plays an important role in budget allocation. Strategies that allocate more samples to $S_{4}$ generally achieve better results. In contrast, assigning more samples to $S_{3}$, such as [5,3,4], can help capture finer details but may also introduce more background clutter, especially in complex scenes such as DroneVehicle. Therefore, simply increasing the sampling budget at the highest-resolution level does not necessarily lead to better detection accuracy.

Among all tested settings, the proposed allocation [4,5,3] achieves the best ${\mathrm{mAP}}_{50}$ on both benchmarks, reaching 85.5% on HIT-UAV and 72.9% on DroneVehicle. Compared with the uniform allocation, this setting slightly increases the sampling budget at $S_{4}$ while reducing redundant sampling at $S_{5}$, leading to a more balanced use of multi-scale features. The consistent improvement on both datasets also indicates that this allocation is not dataset-specific, but has better generalization across different infrared UAV scenarios.

Overall, the ablation results show that HSS improves detection performance without introducing additional computational cost. By adjusting the sampling distribution across feature levels, the model can better match the scale characteristics of infrared targets, which makes the strategy suitable for efficient UAV-based infrared object detection.

### 4.5. Robustness and Performance Boundary Analysis

In practical UAV detection, image degradation such as low signal-to-noise ratio (SNR), motion blur, illumination changes, and adverse weather can greatly affect detection performance. This issue is more severe in long-range infrared scenarios, where UAV targets often occupy only a few pixels. Minor noise or blur may weaken target contours, local textures, and thermal cues, leading to missed detections, false alarms, or inaccurate localization. To examine the robustness boundary of the proposed method, we conduct progressive degradation experiments with noise and blur.

**Figure 14:**
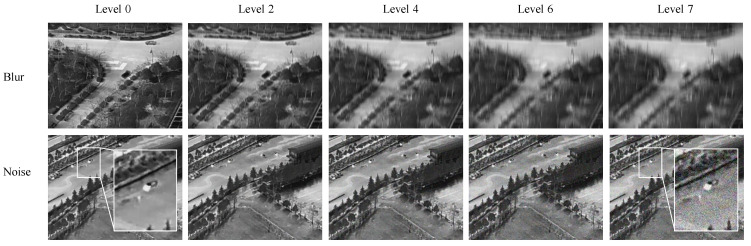
Representative examples under different degradation levels. The top row shows progressive blur degradation, and the bottom row shows progressive noise degradation. Stronger degradation weakens the edge, texture, and local contrast of small targets, increasing detection difficulty.

Progressive noise and blur are added to the HIT-UAV test set. The degradation level is defined from Level 0 to Level 7, where Level 0 denotes the original image, and each higher level increases the perturbation strength by about 5%. Noise degradation simulates low SNR, sensor noise, rain, fog, and other harsh imaging conditions. Blur degradation simulates platform vibration, fast target motion, or exposure mismatch. Representative examples are shown in Figure 14. As degradation increases, the edges and local contrast of small targets gradually fade. Under heavy noise, random bright background responses may resemble infrared UAV targets, while strong blur stretches or diffuses the target contour, making localization less reliable.

**Figure 15:**
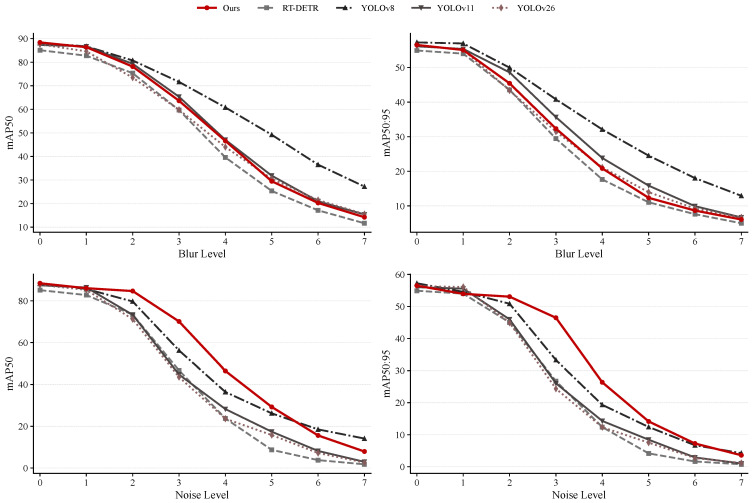
Performance degradation curves of different models on the HIT-UAV dataset under progressive noise and blur. A higher degradation level indicates stronger perturbation.

Figure 15 shows the degradation curves of different models under noise and blur. The mAP of all detectors decreases as the degradation level increases, confirming that strong noise and blur are key failure factors for UAV detection. Under noise perturbation, the proposed method remains relatively stable from Level 0 to Level 5. At Noise Level 3, it achieves 70.11% mAP50 and 46.47% mAP50:95, outperforming most competing methods. At Noise Level 5, it still retains 29.26% mAP50, showing acceptable robustness under moderate low-SNR conditions. However, at Level 6 and Level 7, the target response is largely buried in background noise, and the performance drops sharply, indicating that the model is close to its limit under extremely low SNR.

Under blur degradation, the performance decline is also clear. As the Blur Level increases from 0 to 7, the mAP50 decreases from 88.40% to 14.23%, and the mAP50:95 decreases from 56.50% to 6.08%. Severe blur damages small-target boundaries and spatial cues, so the detector may fail to produce accurate boxes even when it captures a possible target region. Under mild and moderate blur, the proposed method still performs competitively. At extreme blur levels, the performance gap between models becomes smaller, suggesting that visual feature-based detectors share a similar limitation once the target structure is largely lost.

These results show that the proposed method is robust under normal imaging conditions and moderate noise degradation, but its performance decreases rapidly when useful target information is heavily weakened. The main boundary lies in cases where the target-background contrast becomes extremely low, or where severe blur removes the target edges and local textures. In such cases, reliable single-frame detection becomes difficult. Future work may further improve robustness by using temporal information, multi-frame enhancement, image restoration, or uncertainty modeling.

### 4.6. Visualization and Analysis

#### 4.6.1. Feature Visualization

**Figure 16:**
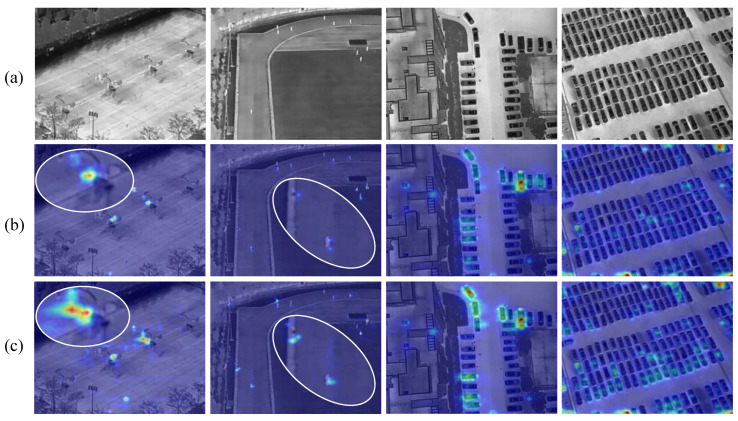
Feature visualization results. The first two groups are from HIT-UAV, and the last two groups are from DroneVehicle. (**a**–**c**) denote the original images, RT-DETR, and EfficientIR-Det, respectively. In the activation maps, warmer colors represent higher response intensity, whereas cooler colors represent lower response intensity.

Figure 16 shows the Grad-CAM activation maps of RT-DETR and EfficientIR-Det. Red regions indicate stronger responses, while blue regions indicate weaker responses. The first two groups are from the HIT-UAV dataset, and the last two groups are from the DroneVehicle dataset.

For the HIT-UAV cases, the first group contains blurred infrared targets, where RT-DETR mainly activates coarse and scattered regions. In contrast, EfficientIR-Det produces more concentrated responses around the target area, showing better alignment with the object location. The second group focuses on small-object detection. Since the targets occupy only a few pixels, accurate localization is difficult. EfficientIR-Det generates sharper and more compact activation regions, while RT-DETR shows weaker and less stable responses around the targets.

For the DroneVehicle cases, the scenes contain more complex top-view backgrounds, such as roads, buildings, and dense parking areas. RT-DETR tends to produce dispersed activations and may respond to background structures with similar thermal patterns. EfficientIR-Det shows more focused responses on vehicle regions, especially in dense or cluttered scenes, indicating better suppression of irrelevant background responses.

Overall, the visualization results suggest that EfficientIR-Det can produce more compact and target-aligned feature responses on both HIT-UAV and DroneVehicle. This also supports its robustness across different infrared UAV detection scenarios.

**Figure 17:**
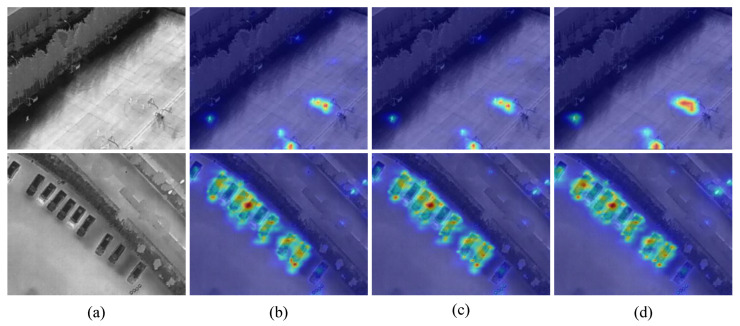
Feature visualization results of different scanning settings on oblique-view infrared scenes: (**a**) original image, (**b**) HiMamba-4way, (**c**) HiMamba-2way, and (**d**) EfficientIR-Det. In the activation maps, warmer colors represent higher response intensity, whereas cooler colors represent lower response intensity.

In addition, Figure 17 provides a visual comparison of different scanning settings on oblique-view infrared scenes. The second and third columns show the activation maps of HiMamba-4way and HiMamba-2way, respectively, and the last column presents the response map of the complete EfficientIR-Det model. In the first scene, where several targets are sparsely distributed along an oblique direction, HiMamba-2way produces activation regions close to those of HiMamba-4way, with the main responses still located around the target areas. In the second scene with densely arranged inclined targets, the two scanning settings also show similar response patterns, and no obvious missing activation is ob served in the HiMamba-2way result.

#### 4.6.2. Detection Performance

**Figure 18:**
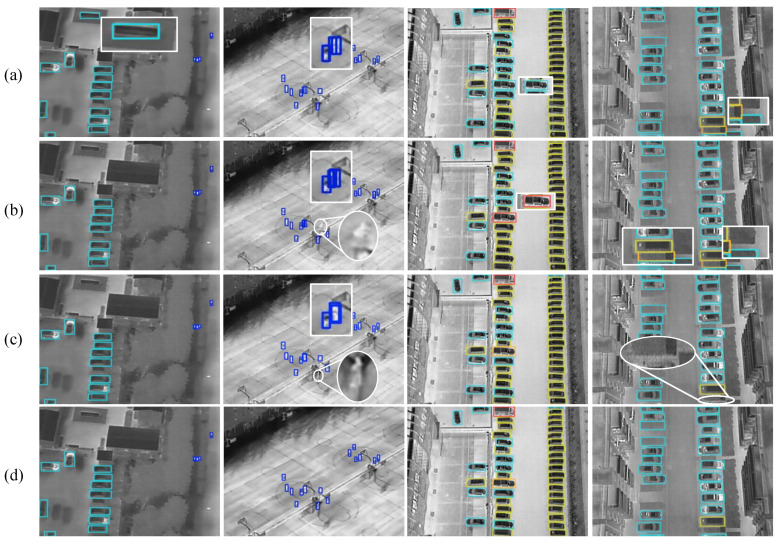
Prediction results on representative scenes. The first two scenes are from HIT-UAV, and the last two are from DroneVehicle. (**a**–**d**) denote RT-DETR, DINO, YOLOv11m, and EfficientIR-Det, respectively. Rectangular annotations indicate false positives or duplicate detections, while circular annotations indicate missed detections.

To provide a qualitative comparison, we visualize the prediction results of RT-DETR, DINO, YOLOv11m, and EfficientIR-Det on four representative scenes, as shown in Figure 18. The first two scenes are from HIT-UAV, and the last two are from DroneVehicle. Rectangular annotations indicate false positives or duplicate detections, while circular annotations indicate missed detections.

In the first HIT-UAV scene, RT-DETR gives a false detection on a background region, whereas the other methods avoid this error. The second scene contains dense small infrared targets. RT-DETR and DINO produce duplicate boxes around several targets, and DINO and YOLOv11m miss one tiny object. EfficientIR-Det produces more compact predictions in this case, with fewer redundant boxes and no obvious omission.

For the DroneVehicle scenes, the third column shows a dense vehicle scenario. RT-DETR and DINO produce false detections, as marked by the white rectangles, which suggests that adjacent vehicles or background structures may be confused with valid targets. YOLOv11m and EfficientIR-Det provide more accurate predictions in this scene. In the fourth column, several targets are located near the image boundary. RT-DETR, DINO, and YOLOv11m all show missed or incorrect detections in the boundary region, while EfficientIR-Det preserves more complete predictions for these edge targets.

These examples show that EfficientIR-Det produces cleaner detection results on both HIT-UAV and DroneVehicle. Compared with the other methods, it is less affected by background interference, dense target distribution, and boundary truncation.

## 5. Conclusions

In this work, we address the accuracy–efficiency trade-off in UAV-based infrared object detection by proposing EfficientIR-Det, a lightweight end-to-end detection framework. To handle the weak texture and low signal-to-noise characteristics of infrared UAV images, we introduce the following four key designs: the Partial Star Network (PSN) backbone for enhancing weak target representations, the Hierarchical Mamba (HiMamba) encoder for efficient global context modeling, and the Adaptive Gated Sampling (AGS) module combined with the Hierarchical Sampling Strategy (HSS) for more effective cross-scale feature fusion and sampling budget allocation.

Experimental results on HIT-UAV and DroneVehicle verify the effectiveness and generalization ability of the proposed method. Compared with the baseline RT-DETR-R18, EfficientIR-Det improves mAP@0.5 by 3.3 pp, reaching 88.4% on HIT-UAV, while reducing parameters and FLOPs by 44.2% and 48.9%, respectively. The framework also maintains a high throughput of 144.7 FPS, outperforming Transformer-based counterparts in real-world inference speed. These results indicate that selective state-space modeling and partial star-shaped computation can improve infrared target representation while keeping the model efficient enough for resource-constrained UAV platforms.

Despite these improvements, some limitations remain. The current sampling budget still relies on heuristic settings, and the scanning patterns in the SSM core are relatively simplified. Future work will focus on adaptive sampling, more stable multi-directional state-space modeling, and evaluation on broader infrared UAV detection scenarios.

## Figures and Tables

**Table 1:** Implementation details of the experimental setup.

Configuration	Details
Operating System	Ubuntu 22.04.1 LTS
GPU	NVIDIA GeForce RTX 3090 (24 GB)
CPU	3.7 GHz, 14-core
Framework	PyTorch 2.2.2 + CUDA 11.2
Optimizer	AdamW
Learning Rate	$1\times 10^{-4}$
Momentum	0.9
Training Epochs	300
Batch Size	16
Input Resolution	640×640
Inference Environment	TensorRT 10.13.3 (FP16)

**Table 2:** Comparison with CNN-based and DETR-based detectors on the HIT-UAV dataset. Bold and underline indicate the best and second-best results, respectively. The upward arrow indicates that higher values are better.

Model	P	R	mAP_0.5_	mAP_0.5:0.95_	AP_*S*_	FLOPs	Params	W.	FPS
	(%)	(%)	(%)	(%)	(%)	(G)	(M)	(MB)	↑
*CNN-based Models*									
RetinaNet [53]	76.3	63.3	74.6	46.4	19.8	82.1	36.4	71.8	67.5
Faster R-CNN [12]	80.1	75.0	78.9	48.9	25.3	91.3	41.8	168.3	55.9
TOOD [54]	84.1	77.8	83.4	53.3	36.1	78.9	32.0	135.6	28.2
SSD [55]	82.3	79.6	82.8	50.3	28.7	137.0	24.1	105.3	66.2
Mamba-YOLO [56]	90.1	78.3	85.4	55.5	42.3	49.6	21.8	49.1	69.4
YOLOv11m [34]	89.2	82.9	87.9	56.1	43.6	67.7	20.0	**26.9**	85.6
YOLOv26m [35]	86.7	81.8	87.6	56.3	**44.7**	67.9	20.3	29.1	**161.0**
*DETR-based Models*									
Swin-DETR [17]	81.1	78.0	82.2	49.9	39.3	147.0	47.8	295.6	84.5
DINO [18]	81.4	81.2	83.1	54.1	33.3	119.0	47.5	313.8	68.9
Deformable-DETR [20]	81.5	79.9	83.2	51.9	39.6	79.6	40.1	281.4	82.9
DDQ [57]	87.3	77.2	84.0	54.2	40.4	115.9	48.3	324.6	52.0
RT-DETR-R18	88.2	84.2	85.1	54.9	43.7	57.0	19.9	43.8	127.1
RT-DETR-L	89.7	83.5	85.5	55.4	43.3	103.4	32.0	48.5	123.0
**Ours: EfficientIR-Det**	**91.4**	**86.1**	**88.4**	**56.5**	44.4	**29.1**	**11.1**	27.9	144.7

**Table 3:** Comparison with CNN-based and DETR-based detectors on the DroneVehicle dataset. Bold and underline indicate the best and second-best results, respectively. The upward arrow indicates that higher values are better.

Model	P	R	mAP_0.5_	mAP_0.5:0.95_	AP_*S*_	FLOPs	Params	W.	FPS
	(%)	(%)	(%)	(%)	(%)	(G)	(M)	(MB)	↑
*CNN-based Models*									
RetinaNet	45.1	58.6	48.5	38.6	10.1	82.3	36.4	285.8	65.1
Tood	65.3	64.4	62.7	39.1	17.6	78.9	32.0	247.2	30.4
Faster R-CNN	43.8	43.6	38.1	26.8	9.7	90.8	30.7	319.1	58.6
mamba-yolo	68.9	68.3	71.3	51.6	16.1	49.6	21.7	83.5	68.3
YOLOv11m	67.7	70.1	72.9	51.2	19.6	67.7	20.0	38.6	103.2
YOLOv8m	67.8	69.3	70.6	50.9	16.6	78.7	25.8	**29.7**	97.5
YOLOv26m	72.8	72.3	**75.2**	54.8	**20.2**	67.9	20.3	43.2	**159.3**
*DETR-based Models*									
DDQ	71.0	70.6	70.1	51.3	16.3	115.9	43.6	421.9	48.5
RT-DETR-R18	72.0	65.1	72.4	52.7	12.4	57.0	19.9	77.0	124.7
RT-DETR-L	77.4	71.7	73.4	**55.7**	17.7	103.5	32.0	125.0	116.6
DEIM	77.4	72.5	73.7	52.3	19.3	90.0	30.7	117.8	105.2
**Ours: EfficientIR-Det**	**77.8**	**74.8**	74.1	55.4	19.7	**29.1**	**11.1**	41.4	140.8

**Table 4:** Incremental component analysis of EfficientIR-Det on the HIT-UAV dataset. Bold indicates the best results. The upward arrow indicates that a higher value is better. The checkmark indicates that the corresponding component is used, while the dash indicates that it is not used.

Model	PSN	HiM	AGS	HSS	P	R	mAP_0.5_	mAP_0.5:0.95_	FLOPs	FPS
					(%)	(%)	(%)	(%)	(G)	↑
Base	–	–	–	–	88.2	84.2	85.1	54.9	57.0	127.1
+PSN	✓	–	–	–	90.4	85.1	86.4	55.1	**25.4**	132.8
+HiM	–	✓	–	–	89.0	82.5	85.1	55.1	57.7	141.6
+AGS	–	–	✓	–	88.9	85.2	86.0	55.9	56.9	124.8
+HSS	–	–	–	✓	88.2	84.3	85.5	55.3	56.9	127.4
+PSN + HiM	✓	✓	–	–	90.5	84.9	86.7	56.0	26.2	**148.8**
+PSN + HiM + AGS	✓	✓	✓	–	91.0	85.9	88.0	56.0	29.1	143.7
+PSN + HiM + AGS + HSS	✓	✓	✓	✓	**91.4**	**86.1**	**88.4**	**56.5**	29.1	144.7

**Table 5:** Incremental component analysis of EfficientIR-Det on the DroneVehicle dataset. Bold indicates the best results. The upward arrow indicates that a higher value is better. The checkmark indicates that the corresponding component is used, while the dash indicates that it is not used.

Model	PSN	HiM	AGS	HSS	P	R	mAP_0.5_	mAP_0.5:0.95_	FLOPs	FPS
					(%)	(%)	(%)	(%)	(G)	↑
Base	–	–	–	–	72.0	65.1	72.4	52.7	57.0	124.7
+PSN	✓	–	–	–	74.2	66.0	73.6	52.9	**25.4**	130.1
+HiM	–	✓	–	–	72.8	64.0	72.4	53.0	57.7	139.3
+AGS	–	–	✓	–	72.7	66.1	73.2	53.5	56.9	123.9
+HSS	–	–	–	✓	72.0	65.2	72.9	53.1	56.9	127.2
+PSN + HiM	✓	✓	–	–	76.8	72.5	73.7	54.8	26.2	**147.4**
+PSN + HiM + AGS	✓	✓	✓	–	77.4	74.0	73.9	55.0	29.1	142.3
+PSN + HiM + AGS + HSS	✓	✓	✓	✓	**77.8**	**74.8**	**74.1**	**55.4**	29.1	140.8

**Table 6:** Comparison of different backbones for RT-DETR on the HIT-UAV dataset. Bold indicates the best results. The upward arrow indicates that a higher value is better.

Backbone Module	P	R	mAP_0.5_	mAP_0.5:0.95_	FLOPs	Params	Weight	FPS
	(%)	(%)	(%)	(%)	(G)	(M)	(MB)	↑
RT-DETR-R18	88.2	84.2	85.1	54.9	57.0	19.9	43.8	127.1
RT-DETR-L	89.7	83.5	85.5	55.4	103.4	32.0	48.5	123.0
+FasterNet [40]	85.3	80.9	83.4	52.8	28.5	10.8	**18.4**	125.6
+EfficientViT [58]	85.7	78.6	82.8	52.1	27.2	10.7	18.5	116.3
+MobileNetV4 [38]	82.5	71.8	85.3	53.1	39.5	11.3	19.5	109.5
+LSNet [59]	87.0	78.6	82.5	51.9	41.1	19.6	42.6	107.8
+StarNet [23]	89.7	81.3	82.3	52.3	31.8	11.9	44.0	120.2
**+PSN (Ours)**	**90.4**	**85.1**	**86.4**	**55.1**	**25.4**	**9.6**	21.5	**132.8**

**Table 7:** Comparison of RT-DETR-R18 variants with different encoder configurations on the HIT-UAV dataset. Bold indicates the best results. The upward arrow indicates that a higher value is better.

Model	P	R	mAP_0.5_	mAP_0.5:0.95_	FLOPs	Params	Weight	FPS
	(%)	(%)	(%)	(%)	(G)	(M)	(MB)	↑
RT-DETR-R18-w/o AIFI	86.0	80.3	83.9	54.2	**56.5**	**19.0**	**41.92**	**151.7**
RT-DETR-R18-AIFI	88.2	**84.2**	**85.1**	54.9	57.0	19.9	43.80	127.1
RT-DETR-R18-HiMamba-4way	88.9	82.2	84.9	**55.1**	57.7	20.8	46.1	131.6
RT-DETR-R18-HiMamba	**89.0**	82.5	**85.1**	**55.1**	57.7	20.7	46.08	147.7

**Table 8:** Comparison of different smoothing and sampling methods on object detection performance on the HIT-UAV dataset. Bold indicates the best results.

Method	P	R	mAP_0.5_	mAP_0.5:0.95_
	(%)	(%)	(%)	(%)
Sampling (Base)	90.5	84.9	86.7	55.1
SBA [60]	86.8	83.4	86.0	54.4
WaveletPool [61]	88.9	79.4	82.7	52.1
wConv [62]	89.8	79.3	85.1	53.5
GCCconv [63]	88.6	80.7	84.8	54.7
**AGS (Ours)**	**91.0**	**85.9**	**88.0**	**56.0**

**Table 9:** Sampling allocation strategies at different feature scales (S3, S4, S5) on the HIT-UAV and DroneVehicle datasets. Bold indicates the best results.

S3	S4	S5	HIT-UAV		DroneVehicle	
			mAP_0.5_(%)	mAP_0.5:0.95_ (%)	mAP_0.5_(%)	mAP_0.5:0.95_ (%)
3	4	5	82.4	53.9	72.4	52.4
3	5	4	83.6	54.3	72.8	52.9
4	3	5	81.6	52.6	71.9	52.3
**4**	**5**	**3**	**85.5**	55.0(−0.2)	**72.9**	**54.1**
4	4	4	85.1	55.0	72.4	52.7
5	3	4	85.0	55.1	72.7	53.0
5	4	3	84.7 (−0.8)	**55.2**	72.5	52.8

## Data Availability

The HIT-UAV dataset used in this study is publicly available at https://github.com/suojiashun/HIT-UAV-Infrared-Thermal-Dataset (accessed on 18 October 2025).
